# Advances in hemiplegia rehabilitation: modern therapeutic interventions to enhance activities of daily living

**DOI:** 10.3389/fneur.2025.1555990

**Published:** 2025-03-28

**Authors:** Jing Xiong, Jin-Tian Wang, Shu Lin, Bao-Yuan Xie

**Affiliations:** ^1^Department of Nursing, The Second Affiliated Hospital of Fujian Medical University, Quanzhou, China; ^2^Department of Colorectal Surgery, The Second Affiliated Hospital of Fujian Medical University, Quanzhou, China; ^3^Centre of Neurological and Metabolic Research, The Second Affiliated Hospital of Fujian Medical University, Quanzhou, China; ^4^Group of Neuroendocrinology, Garvan Institute of Medical Research, Darlinghurst, NSW, Australia

**Keywords:** hemiplegia, rehabilitation, therapeutic interventions, activities of daily living, modern rehabilitation techniques

## Abstract

Hemiplegia severely impairs patients’ abilities to perform activities of daily living (ADL), thus affecting their overall quality of life and independence. Often caused by stroke or other forms of brain injury, hemiparesis causes long-term impairment of upper and lower limb function, hindering the patient’s ability to manage self-care. With advances in modern rehabilitation medicine, emerging therapeutic interventions such as electrophysiological feedback, virtual reality, and robot-assisted therapy are increasingly being applied to the rehabilitation of hemiplegic patients. These interventions, combined with precise technical support through individualized training, have been shown to be effective in improving upper and lower limb function as well as enhancing ADL abilities of hemiplegic patients. This paper reviews recent advances in modern hemiplegic rehabilitation therapeutic interventions, assesses their impact on improving ADL performance, and examines their effectiveness in improving functional outcomes and quality of life for patients. These findings suggest that modern rehabilitation approaches have significant clinical potential to provide more personalized and effective treatment strategies for people with hemiplegia.

## 1 Introduction

Hemiplegia is a neurological condition caused by stroke, traumatic brain injury, or other neurological damage, usually manifesting as weakness or paralysis on one side of the body (1). It causes significant motor impairment, spasticity, imbalance, and cognitive deficits, severely limiting a patient’s ability to perform activities of daily living (ADL) (2). The loss of ADL function not only affects patient independence but also imposes a substantial burden on families and society (3). Traditional rehabilitation methods, such as physical therapy (PT), occupational therapy (OT), and pharmacological interventions, aim to restore function through motor training and neuromuscular stimulation (4). While these approaches offer benefits, their effectiveness varies, particularly in patients with severe impairments, leading to increasing interest in more advanced rehabilitation strategies (5, 6).

With the continuous advancements in rehabilitation medicine, an increasing number of technologies have been integrated into functional recovery strategies for hemiplegic patients. Among these modern rehabilitation approaches, FES, RAT, and VR have emerged as three prominent interventions. FES has been a well-established neuromuscular rehabilitation technique for decades. However, its integration with RAT and VR in recent years has further expanded its clinical applications and enhanced therapeutic efficacy (7). RAT provides controlled, repetitive motor support, while VR-based interventions encompassing both immersive and non-immersive approaches offer engaging, task-specific rehabilitation to enhance motor and cognitive function. Despite promising advancements, challenges remain, including long-term effectiveness, patient adaptability, cost, and integration into standard rehabilitation protocols.

This review was conducted as a narrative literature review, focusing on the role of VR, RAT, and FES in improving ADL recovery in hemiplegic patients. Relevant studies were identified through PubMed, Scopus, and Web of Science, covering publications from 2015 to 2024. The selection prioritized peer-reviewed articles, including systematic reviews, meta-analyses, randomized controlled trials (RCTs), and cohort studies, which investigated the effects of these rehabilitation technologies. Studies without relevant outcome data or non-clinical studies were excluded to ensure the focus remained on clinical applicability.

This narrative review critically evaluates the application and effectiveness of VR, RAT, and FES in improving ADL recovery in hemiplegic patients. It assesses their benefits, limitations, and clinical applicability, while also identifying existing challenges and research gaps. Furthermore, it explores strategies to optimize these rehabilitation technologies for broader clinical adoption and future research development.

## 2 Overview and historical development of hemiplegia rehabilitation

The development of hemiplegia rehabilitation has spanned the evolution from understanding its definition and causes to treatment approaches. Hemiplegia due to damage to the central nervous system results in paralysis on one side of the body and significantly affects the patient’s ability to perform ADL. Traditional rehabilitation approaches, including physical therapy, occupational therapy, speech therapy, and pharmacological interventions, have long been utilized. However, these methods have notable limitations in fully addressing the complex needs of ADL recovery, particularly with regard to individualization and cognitive challenges. This section outlines the historical background of hemiplegia rehabilitation, setting the stage for the introduction of modern therapeutic interventions.

### 2.1 Hemiplegia: definition and causes

**Figure 1:**
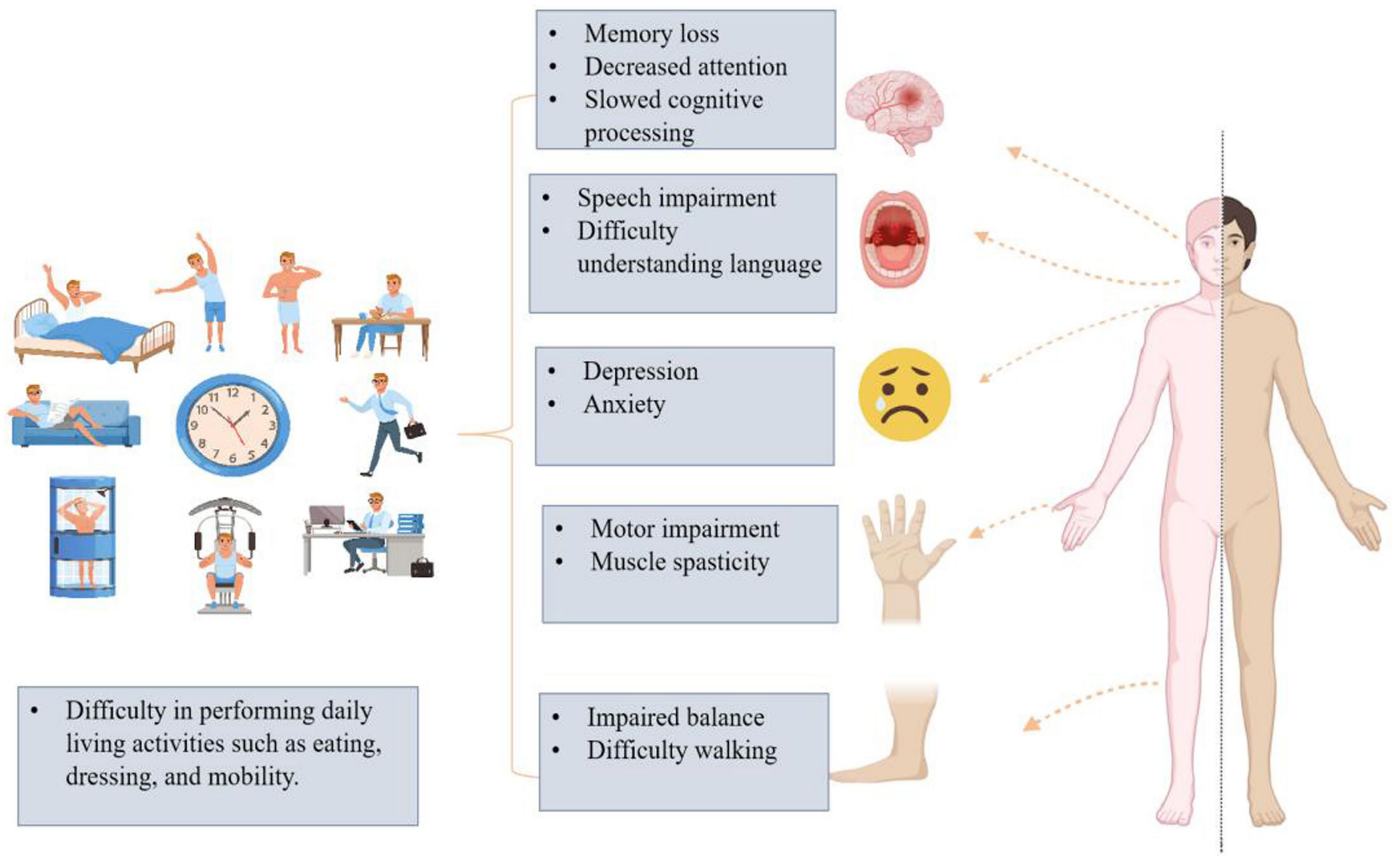
Diagram shows the multifaceted impacts of hemiplegia on patients’ cognitive, emotional, and physical functions, and how these impairments restrict activities of daily living. From a cognitive perspective, hemiplegia can lead to memory loss, decreased attention, and slowed cognitive processing. In terms of language, patients may experience speech impairments and difficulty understanding language. Emotionally, hemiplegia is usually accompanied by depression and anxiety. Physically, it results in motor impairment, muscle spasticity, and balance disorders, leading to difficulties in mobility. These impairments severely affect patients’ ability to perform basic daily activities such as eating, dressing, and walking.

Hemiplegia is partial or complete paralysis of one side of the body, usually due to damage to the motor pathways of the brain (8). This neurological condition usually follows a stroke, traumatic brain injury (TBI), or other central nervous system disorders that impair motor control on one side of the body (9). Stroke, either ischemic or hemorrhagic, remains the most prevalent cause of hemiplegia, accounting for a large proportion of cases globally (10). Ischemic stroke occurs when blood flow to a part of the brain is blocked (usually due to a blood clot), whereas hemorrhagic stroke is caused by bleeding in or around the brain, leading to localized brain damage (11). Other causes include traumatic brain injuries from accidents, tumors affecting brain regions responsible for motor control, and infections such as encephalitis or meningitis (12). These conditions lead to a loss of voluntary control of the muscles on the affected side of the body, impairing patients’ ability to perform ADL. The impacts of hemiplegia extend beyond muscle weakness or paralysis. Patients often suffer from spasticity, poor coordination, and sensory deficits on the affected side, further limiting their mobility and independence (13). Additionally, because hemiplegia disrupts critical neural pathways, it can lead to secondary conditions such as joint deformities, contractures, and pressure sores, especially if not managed with appropriate rehabilitation interventions (Figure 1).

### 2.2 Traditional hemiplegia rehabilitation approaches

**Figure 2:**
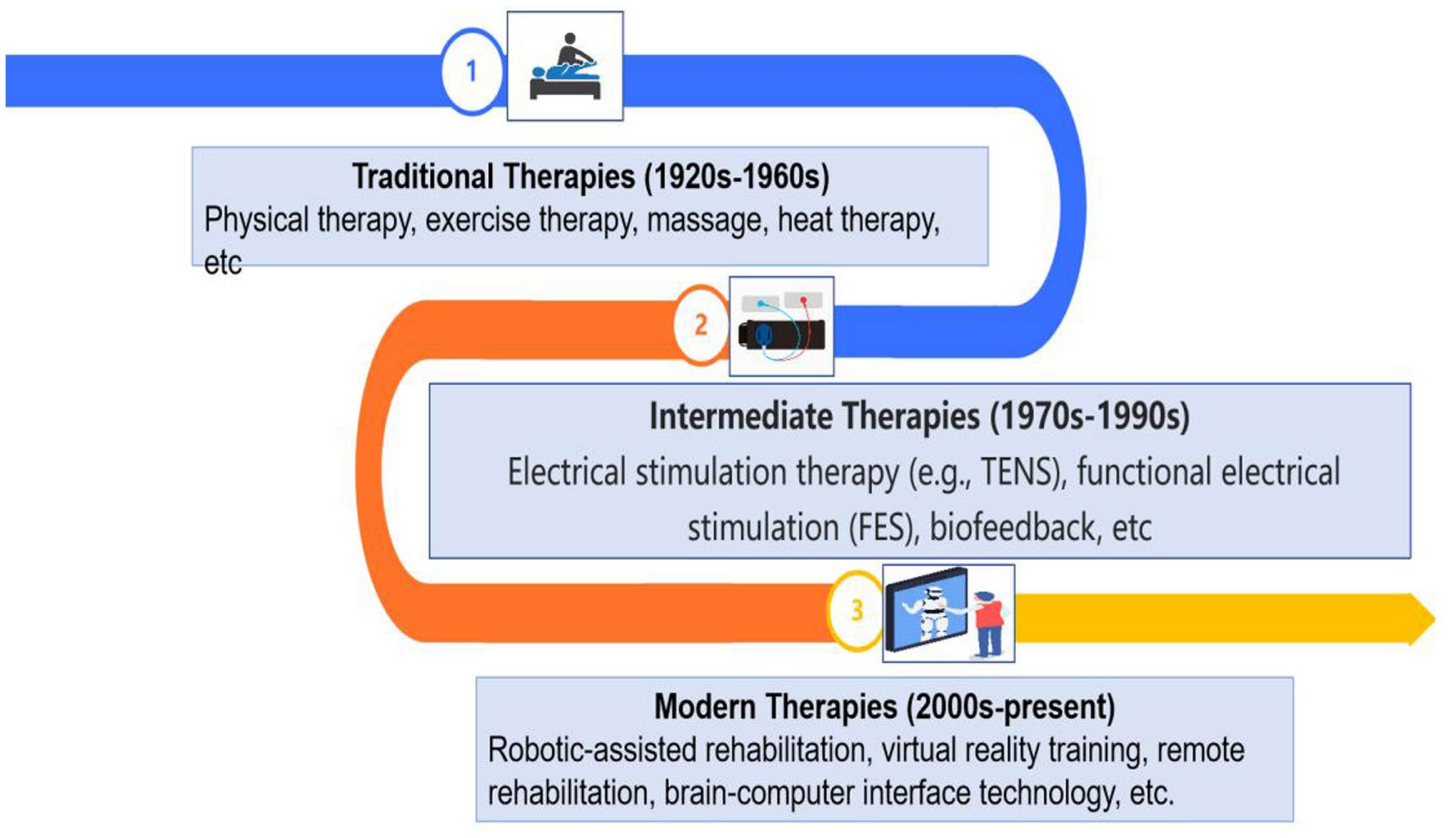
Diagram shows the evolution of hemiplegia rehabilitation methods, divided into three key phases. In the 1920s–1960s, traditional therapies mainly relied on physical therapy, exercise therapy, and massage, utilizing manual techniques and patient-driven movement to promote recovery. Next, in the 1970s–1990s, intermediate therapies introduced electrical stimulation therapies (e.g., TENS) and functional electrical stimulation (FES), which helped muscles recover through electrical signals, significantly improving rehabilitation outcomes. In the 2000s to the present, modern therapies emerged, including robotic-assisted rehabilitation, virtual reality training, and remote rehabilitation. These advanced technologies have made rehabilitation more precise and personalized, greatly shortening recovery time and improving effectiveness. This evolution demonstrates how technological advances are driving the development and improvement of rehabilitation methods.

Traditional hemiplegia rehabilitation approaches mainly include physical therapy, occupational therapy, speech therapy, and pharmacological interventions. These approaches have been practiced for decades and are widely used to promote functional recovery in patients with hemiplegia (Figure 2).

#### 2.2.1 Physical therapy

Physical therapy (PT) is one of the most common methods in hemiplegia rehabilitation. It aims to restore impaired motor functions through exercise training, balance training, and muscle strengthening (14). Physical therapists typically design individualized training programs for patients based on their specific conditions, including gait training, range of motion exercises, and postural control training (15). The core principle of physical therapy is to stimulate neuroplasticity in the brain through repetitive movements, leading to partial functional recovery (16).

However, PT primarily focuses on motor function improvement in controlled settings, often failing to address the challenges patients face in real-world activities. To bridge this gap, patient education has been integrated into rehabilitation, incorporating problem-solving discussions to help patients adapt to uneven surfaces, crowded environments, and daily obstacles (17).

While these educational strategies support functional transfer, behavior-oriented approaches such as constraint-induced movement therapy (CIMT) and behavioral change therapy (BCT) offer additional methods to enhance long-term adherence and engagement, which will be discussed in section 2.4.

#### 2.2.2 Occupational therapy

The primary goal of occupational therapy (OT) is to increase the patient’s independence in ADL by focusing on fine motor skills training, such as hand gripping and object manipulation (18, 19). Occupational therapists assist patients relearn these skills by simulating real-life scenarios (e.g., dressing, eating, and writing). Although OT can enhance a patient’s ability to perform everyday tasks, its limitation is the simulated environment, which may not always translate effectively to real-world situations (20).

#### 2.2.3 Speech therapy

For hemiplegic patients suffering from language disorders such as aphasia or dysphagia, speech therapy (ST) is a critical component of the traditional rehabilitation process (80). Speech therapy aims to help patients regain their ability to communicate by retraining speech articulation, grammar, and vocabulary (21). If the patient experiences dysphagia, targeted swallowing exercises will be used as part of the rehabilitation plan (22).

#### 2.2.4 Pharmacological interventions

Pharmacological treatments are commonly used to control spasticity, muscle stiffness, and pain in hemiplegic patients (23). Commonly used medications include muscle relaxants (e.g., baclofen), antispastic agents, and anticonvulsants (24). These medications can temporarily improve muscle condition and enable patients to better participate in physical and occupational therapy. However, prolonged use of these medications may lead to dependence and may not address the underlying cause of motor dysfunction.

### 2.3 Challenges of traditional rehabilitation approaches

Although traditional rehabilitation approaches (e.g., PT, OT, and pharmacologic interventions) are fundamental to the treatment of hemiplegia, they face significant limitations in fully restoring function, particularly in the area of ADL. Understanding these challenges highlights the need for more advanced therapeutic interventions (Table 1).

**Table 1:** This table summarizes the comparison of characteristics, applicability, and accessibility of different rehabilitation methods.

Rehabilitation methods	Advantages	Disadvantages	Applicable population	Cost	Accessibility	Source
Physical therapy (PT)	Proven effective method; low technical requirements; suitable for a wide range of patients	Slow progress; requires long-term treatment; depends on professional guidance	Patients with various types of hemiplegia	Low, depends on treatment frequency	High, offered by most medical institutions	(54)
Occupational therapy (OT)	Focused on restoring daily activities; personalized treatment; helps improve independence	Effectiveness depends on patient cooperation; requires intensive therapy	Patients with limited ability to perform daily activities	Moderate, depends on treatment frequency	High, available at many rehabilitation centers	(18)
Speech therapy (ST)	Improves speech ability and communication skills; enhances daily communication	Long treatment duration; some patients may see limited results	Patients with speech impairments and swallowing difficulties	Moderate, depends on treatment frequency	High, available at many rehabilitation centers	(21)
Pharmacological interventions	Effectively reduces spasticity, pain, and depressive symptoms; serves as an adjunct in rehabilitation	May have side effects; requires long-term medication; cannot solve functional impairments alone	Patients with severe spasticity or emotional disorders	Moderate to high, depending on medication type and treatment duration	High, widely used in various medical institutions	(23)
Virtual reality technology (VR)	Provides immersive rehabilitation training environment; enhances patient engagement; increases opportunities for self-training	High dependence on technology; equipment is expensive; some patients may not adapt well	Younger patients or those more accepting of technology	High, expensive equipment	Moderate, mainly accessible in developed regions and large rehabilitation centers	(35)
Robotic-assisted rehabilitation	Precise motor control; reduces the workload of rehabilitation staff; effective for repetitive training	Expensive equipment; complex maintenance; limited applicability	Patients with moderate to severe hemiplegia; those with severe motor impairments	Very high, expensive equipment	Low, only available at large rehabilitation centers	(45)
Functional electrical stimulation (FES)	Stimulates neuromuscular recovery; suitable for different recovery stages	Requires patient cooperation; some patients may find it uncomfortable; not suitable for all patients	Patients with partial motor function loss	Moderate, depends on equipment and frequency of use	Moderate, equipment is becoming more widespread but not yet widely available	(51)

#### 2.3.1 Limited functional gains in ADL recovery

One of the main challenges with traditional rehabilitation approaches is their limited impact on restoring full functional independence in ADL. While therapies such as PT and OT can improve basic motor function, they often fail to fully replicate the complex motor and cognitive coordination required for daily activities. ADL tasks such as dressing, eating, and personal hygiene require not only physical movement, but also higher levels of cognitive integration, which may not be adequately addressed by traditional approaches (25). This limitation is particularly evident in patients with severe movement disorders, whose progress in functional recovery tends to stabilize after a certain stage of rehabilitation.

#### 2.3.2 Lack of individualization and precision

Traditional rehabilitation therapies are usually generalized and less often tailored to the specific needs of individual patients. Despite the design of individualized treatment plans, the nature of traditional approaches often lacks the precision needed to target specific neural pathways or muscle groups. For instance, physical therapy relies heavily on repetitive movements that may not always engage the necessary neural circuits required for optimal recovery. Additionally, traditional approaches may fail to take into account the specific cognitive or emotional impairments that each patient faces during rehabilitation, limiting their overall effectiveness (26).

#### 2.3.3 Insufficient engagement and motivation

Traditional rehabilitation exercises can become monotonous, making it difficult for patients to stay motivated and engaged, particularly younger individuals and those with cognitive impairments (27). While repetitive task training is fundamental to rehabilitation, solely relying on it can lead to decreased adherence. To address this, behavioral change therapy (BCT) integrates goal setting, behavioral contracting, and problem-solving discussions to enhance engagement and long-term participation (17).

Given these challenges, incorporating behavior-oriented strategies such as BCT and constraint-induced movement therapy (CIMT) may improve patient motivation and adherence, as further discussed in section 2.4.

#### 2.3.4 Plateau in long-term progress

A common issue in traditional rehabilitation is the plateau effect, where patients reach a stage in their recovery where further progress becomes minimal or stagnant. This plateau can occur due to the limitations of traditional methods in promoting neuroplasticity (the ability of the brain to reorganize and form new neural connections). Since neuroplasticity plays a crucial role in recovery, especially after a stroke, traditional therapies may not adequately stimulate the brain to continue the healing process beyond a certain point. As a result, many patients lack significant long-term improvements in motor function and ADL independence (28).

#### 2.3.5 Limited integration of cognitive and motor rehabilitation

Traditional approaches to rehabilitation focus primarily on the motor or cognitive aspects of rehabilitation, often viewing them as separate components. However, successful ADL recovery requires an integrated approach that combines motor function rehabilitation with cognitive training. For example, tasks such as cooking involve motor skills and cognitive planning. The lack of integration in traditional therapies may lead to incomplete recovery, as patients may improve physically but still face cognitive deficits that limit their overall independence (29).

### 2.4 Behavior-oriented rehabilitation approaches

Traditional rehabilitation primarily targets motor function recovery in controlled environments, but its real-world effectiveness is often limited by habitual nonuse of the affected limb, poor long-term adherence, and decreased patient motivation (17, 30). Behavior-oriented rehabilitation strategies, particularly constraint-induced movement therapy (CIMT) and behavioral change therapy (BCT), aim to enhance patient engagement and functional independence, thereby improving long-term rehabilitation outcomes.

#### 2.4.1 Constraint-induced movement therapy

CIMT, rooted in neuroplasticity theory, is designed to counteract learned nonuse by restricting the unaffected limb and compelling patients to engage the affected side in high-intensity, task-oriented training (≥6 h/day). In chronic stroke patients, CIMT has been shown to increase affected limb use fivefold within 2 weeks, with effects persisting beyond 2 years (30). Neuroimaging studies further support its role in cortical reorganization, expanding motor cortex representation and increasing gray matter density in the affected hemisphere (31, 32).

#### 2.4.2 Behavioral change therapy

BCT, based on social cognitive theory (SCT), focuses on sustaining patient adherence through structured interventions such as goal setting, therapist-guided activity tracking, and remote monitoring. A multicenter RCT demonstrated that BCT increased moderate-to-vigorous physical activity (MVPA) by 42% over 6 months, with adherence maintained at 12-month follow-up (17). Moreover, BCT significantly reduced dropout rates compared to standard physical therapy (5% vs. 23%), underscoring its role in enhancing long-term patient engagement.

#### 2.4.3 Integration with traditional and modern rehabilitation

Unlike traditional rehabilitation, which primarily focuses on task completion, behavior-oriented rehabilitation emphasizes the sustained use of recovered motor functions in real-world contexts. These behavioral strategies can be integrated into conventional therapy (e.g., occupational therapy with home-based assignments) or modern rehabilitation frameworks (e.g., adherence management in telerehabilitation), ultimately enhancing functional recovery beyond the clinical setting.

## 3 Modern therapeutic interventions and ADL recovery

In recent years, significant progress has been made in hemiplegia rehabilitation with the advancement of modern technologies such as virtual reality (VR), robot-assisted therapy (RAT), and functional electrical stimulation (FES). These interventions not only contribute to motor function recovery but also offer personalized training programs aimed at improving ADL independence and quality.

While studies have demonstrated the effectiveness of these technologies in structured clinical and laboratory settings, their impact on real-world, spontaneous physically-based activity remains less clear. Many rehabilitation programs report high levels of patient engagement and functional improvements within controlled environments, yet evidence on their long-term translation into daily life remains limited. Patients often face challenges in maintaining the same level of activity and independence outside supervised therapy sessions, highlighting the need for strategies that reinforce sustained ADL engagement beyond structured rehabilitation (33).

This section will explore how these modern technological interventions interact with each other in hemiplegia rehabilitation and critically evaluate their role in facilitating ADL recovery, considering both their benefits and their limitations in real-world application (Table 2).

**Table 2:** This table clearly summarizes modern therapeutic interventions for hemiplegia rehabilitation, including virtual reality (VR), robot-assisted therapy (RAT), functional and electrical stimulation (FES).

Modern therapeutic interventions	ADL score improvement	Muscle strength improvement	Motor control recovery	Other therapeutic indicators	Source
Virtual reality (VR)	Improvement 10.05 (*p* < 0.001)	Not mentioned	Improvement 3.73 (*p* = 0.0004)	Significant improvement in balance ability (*p* < 0.001)	(5)
Virtual reality (VR)	Not mentioned	Not mentioned	Not mentioned	Alleviated shoulder pain, enhanced rehabilitation effectiveness	(79)
Virtual reality (VR)	Significant ADL improvement	Upper limb function and control improved	Enhanced engagement and accuracy	Increased patient participation and balance	(34)
Robot-assisted therapy (RAT)	Significant ADL improvement (*p* < 0.001)	Not mentioned	Improved gait symmetry (*p* = 0.044)	Significant improvement in 6-min walk test and FIM-walk	(45)
Robot-assisted therapy (RAT)	Significant improvement in Barthel Index	Upper limb motor function recovery	Significant improvement in Modified Ashworth Scale and ARAT	Post-stroke pain prevention	(44)
Robot-assisted therapy (RAT)	Significant ADL improvement	Increased upper limb strength (improved hand grip)	Improved motor control, increased training intensity	Improved motor function and hand grip strength	(48)
Robot-assisted therapy (RAT)	Increased ADL score, improved upper limb motor function	Improved upper limb motor control	Bilateral upper limb function recovery, significant hand flexibility improvement	Significant recovery of bilateral upper limb function	(46)
Functional electrical stimulation (FES)	MBI improvement (data not provided)	Not mentioned	Limited improvement in spasticity	Partial improvement in spasticity	(4)
Functional electrical stimulation (FES)	Significant ADL improvement (*p* = 0.001)	Significant FMUE improvement (*p* = 0.001)	Significant improvement in upper limb motor function	Significant improvement in Brunnstrom stage (*p* = 0.001)	(51)
Functional electrical stimulation (FES)	Significant improvement in ADL (via HANDS therapy)	FMA-UE improvement (21 points → 28 points)	Not mentioned	MAL-AOU and MAL-QOM improvement	(6)
Functional electrical stimulation (FES)	Significant ADL improvement (Barthel Index)	Improved upper limb strength (fingers, upper limbs)	Improved upper limb motor function	Enhanced hand flexibility, improved daily activity ability	(52)

### 3.1 Virtual reality in ADL enhancement

In recent years, VR technology has been widely used in the rehabilitation of hemiplegia patients (34). By creating virtual environments, VR simulates real-world scenarios, enabling patients to safely participate in ADL training. This technology helps patients gradually restore motor function and improve cognitive and coordination skills, especially in the early stages of rehabilitation (35). Through virtual tasks like dressing and eating, VR improves limb coordination and hand-eye coordination, and studies have shown improvements in Barthel Index (BI) scores, reflecting better self-care abilities (36). However, BI primarily measures assistance levels in basic ADL rather than the quality of task execution. To provide a more comprehensive assessment of VR’s impact, additional outcome measures, such as the functional independence measure (FIM) for assessing cognitive and motor function, the timed up and go (TUG) test for evaluating balance and mobility, and patient-reported outcomes (PROMs) reflecting self-efficacy in ADL, should be considered (37).

Additionally, VR provides a risk-free training environment where patients can repeatedly practice specific movements with real-time feedback, accelerating motor learning and neuroplasticity (38). While VR has proven effective for ADL enhancement, the technology can be applied in two primary forms: immersive and non-immersive VR, each with its unique benefits for different rehabilitation needs. The following section compares these two types of VR and their specific applications in enhancing ADL functions.

#### 3.1.1 Immersive vs. non-immersive VR

VR technology can be applied in different forms to meet diverse patient needs. Immersive VR, which uses head-mounted displays (HMDs) such as Oculus Rift or HTC Vive, creates a fully interactive, multisensory environment. This type of VR enhances motor and cognitive skills through real-time feedback and high engagement. Studies have shown that immersive VR not only improves motor learning but also contributes to better functional independence in ADL, as assessed by measures such as the functional independence measure (FIM) and patient-reported outcome measures (39). The immersive experience strengthens the patients’ involvement, facilitating more effective motor relearning. However, immersive VR systems require specialized equipment, which can be costly and impractical for use in resource-limited clinical settings.

On the other hand, non-immersive VR employs screen-based systems such as motion-tracking devices (e.g., Microsoft Kinect, Nintendo Wii) or traditional computer monitors. While it offers less sensory feedback, non-immersive VR remains a more cost-effective and accessible solution for clinical settings with budget constraints. It is particularly useful for patients with severe impairments or those in the early stages of recovery who may not require the level of interaction provided by immersive VR. Despite its simpler interface, non-immersive VR has demonstrated substantial improvements in motor coordination and balance, which are crucial components of ADL performance (40).

#### 3.1.2 Comparison of effectiveness in ADL rehabilitation

Both immersive and non-immersive VR have demonstrated positive effects in improving ADL functions. However, the degree of effectiveness varies depending on patient characteristics and the specific ADL demands. Immersive VR provides higher levels of engagement, making it better suited for patients who can tolerate more complex interactions and benefit from highly stimulating environments. The multisensory feedback provided by immersive VR enhances motor learning and ADL independence, making it particularly effective for patients with mild to moderate disabilities. On the other hand, non-immersive VR is a more affordable and practical alternative for patients with severe disabilities or those in the early stages of recovery. While it does not offer the same level of engagement as immersive VR, it still delivers significant improvements in motor coordination and balance. This makes it an ideal solution for clinics with limited resources (41, 42). Both VR systems offer individualized training that promotes neuroplasticity and enhances ADL independence, but the choice of system depends on the patient’s condition and available resources.

### 3.2 Robotic-assisted therapy and functional recovery

RAT has emerged as a transformative tool in the rehabilitation of hemiplegic patients, particularly in the recovery of upper and lower limb functions (38). This technology utilizes robotic systems to guide and assist patients through repetitive, controlled movements that mimic natural motions. These robotic devices can be customized for each patient’s specific dysfunction, ensuring targeted treatment. By providing highly precise, adjustable, and repetitive exercises, RAT plays a key role in accelerating motor function recovery and supporting ADL improvement (43). One of the key advantages of RAT is its ability to provide consistent, measurable, and repeatable movements, which are critical in neurorehabilitation. Traditional manual therapy often relies on the strength and expertise of the therapist, which can lead to inconsistencies. In contrast, robotic systems provide uniform assistance with precise force, duration and range of motion, ensuring standardized and effective rehabilitation. Such controlled repetition is particularly useful in neuroplasticity, where repetitive, task-specific movements help retrain the brain to compensate for motor deficits caused by stroke or other brain injuries (44, 45).

RAT is typically used for both upper and lower limb rehabilitation. In upper limb rehabilitation, the robot helps patients regain functional mobility through repetitive tasks such as reaching, grasping, or manipulating objects, essential for self-care activities like dressing and eating. Lower limb rehabilitation focuses on restoring balance, gait, and strength, which are crucial for mobility and self-care. Several studies have shown that the use of robotic-assisted gait training significantly improves walking ability and overall mobility in hemiplegic patients (23, 46). However, while RAT has demonstrated success in clinical and laboratory settings, its impact on real-world self-care spontaneous activities remains less well understood. Many current outcome measures, such as the Barthel Index (BI) and Functional Ambulation Category (FAC), primarily assess assistance levels rather than the quality or frequency of self-initiated ADL engagement. To address this limitation, studies have begun incorporating validated self-report assessments—such as the Motor Activity Log (MAL), which evaluates real-world arm use, and the Stroke Impact Scale (SIS), which measures functional independence beyond clinical settings (47). Additionally, body-worn motion monitors, including accelerometers and step counters, provide objective, continuous monitoring of spontaneous physical activity in everyday environments, offering a more comprehensive evaluation of RAT’s impact on ADL recovery (48).

Studies have reported significant improvements in ADL performance when RAT is combined with other rehabilitation methods, such as functional electrical stimulation (FES) or traditional physical therapy. The combination of these therapies enhances functional recovery by addressing muscular and neurological deficits. For example, patients receiving robotic-assisted gait training, combined with conventional rehabilitation, show greater long-term gains in mobility and ADL independence than those receiving either therapy alone (49, 50).

In summary, robotic-assisted therapy represents a significant advancement in hemiplegia rehabilitation, offering consistent, targeted, and measurable interventions that accelerate functional recovery. However, to maximize its impact on real-world self-care activities, future research should incorporate behavioral rehabilitation strategies (e.g., BCT, CIMT) and integrate real-world activity monitoring tools to assess long-term ADL engagement more comprehensively.

### 3.3 Functional electrical stimulation for muscle re-education

FES is a widely used therapeutic method in neurorehabilitation, particularly for muscle re-education in patients with hemiplegia. FES works by electrically stimulating the neuromuscular system to induce muscle contractions to assist in the restoration of functional movement. This therapy is particularly effective for patients with muscle atrophy or neural damage, and plays a crucial role in enhancing ADL recovery (51). The basic principle of FES involves delivering a mild electric current through electrodes to specific muscle groups, mimicking the signals that the brain normally sends to trigger muscle contractions. For hemiplegic patients, FES helps to restore neuromuscular connections, and repetitive training with FES fosters muscle memory and the re-establishment of motor control. This process is especially important for patients with impaired motor signals due to brain injury (52).

FES has demonstrated significant benefits in improving ADL recovery. For upper limb function, FES can help patients regain the ability to grasp, release, and move their arms, which is critical for performing self-care activities such as eating, dressing, and personal hygiene. In the lower limbs, FES has shown considerable efficacy in helping patients recover gait and balance, particularly in strengthening ankle function and addressing issues like foot drop (53). These improvements have a direct impact on patients’ mobility and ADL performance, enhancing their independence. The benefits of FES in increasing muscle strength, endurance, and control are well-supported by research. A study of hemiplegic patients showed that those who received FES in conjunction with traditional rehabilitation therapy achieved better outcomes in terms of ADL scores (e.g., Barthel Index) and muscle strength tests compared with a control group that did not receive FES (54). By aiding in muscle recovery, FES enables patients to use the affected limbs more effectively in their daily lives and reduce their dependence on external assistance.

FES can also be integrated with other modern therapies, such as RAT or VR technology. The combination of multimodal rehabilitation therapies can have synergistic effects, further enhancing muscle control and neuroplasticity, thus maximizing functional recovery outcomes (55, 56). Despite the potential of FES, challenges remain. Patient compliance and adaptability are key factors in the successful application of FES, especially in long-term treatment. While device availability and cost may impact clinical adoption, FES is generally more accessible than robotic-assisted therapy (RAT) and some advanced VR systems (57). With continued technological advances and improvements in integration with other rehabilitation modalities, FES is expected to further strengthen its role in promoting independence and improving the quality of life in hemiplegic patients.

## 4 Short-term efficacy and long-term sustainability of modern rehabilitation therapies

Modern rehabilitation therapies, such as VR, RAT, and FES, have shown significant short-term results in hemiplegia rehabilitation, particularly in improving motor function and ADL. However, while these therapies are effective in promoting neuroplasticity and functional recovery in the short term, their long-term sustainability and effectiveness remain areas of ongoing research. As the rehabilitation process progresses, challenges such as diminished effectiveness, patient compliance, and high equipment costs pose significant barriers to sustaining long-term benefits. Therefore, this section will explore the short-term efficacy and long-term sustainability of these therapies, providing insights for future research and development of rehabilitation technologies.

### 4.1 Short-term efficacy and functional recovery of multimodal combined therapies

In recent years, multimodal combined therapies have demonstrated significant short-term efficacy in hemiplegia rehabilitation, particularly in the recovery of motor function and ADL. The combination of VR, RAT, and FES produces synergistic effects by targeting motor and cognitive pathways, resulting in more comprehensive rehabilitation outcomes than monotherapy. This section examines the use of these combined therapies in functional recovery of the upper and lower extremities, explores how they enhance neuroplasticity, and highlights the flexibility of individualized rehabilitation programs.

#### 4.1.1 Advantages of multimodal approaches

When used alone, VR, RAT, and FES have shown significant improvements in motor function and ADL recovery. However, a growing body of research suggests that the combined use of these therapies can produce synergistic effects superior to any single therapy. For example, VR offers an immersive environment that enhances cognitive engagement, RAT provides the precise and repetitive training needed for neuroplasticity, and FES stimulates muscle contraction and promotes motor relearning (58). This multimodal approach targets both motor and cognitive pathways, resulting in a more comprehensive rehabilitation process.

#### 4.1.2 Applications in upper and lower limb rehabilitation

Combined therapies have been particularly effective in promoting the recovery of both upper and lower limb functions. For the upper limbs, integrated VR and RAT allow patients to practice real-world tasks, such as reaching and grasping, in a simulated environment, while FES retrains the muscles to contract and perform these movements more effectively (59). In the lower extremities, this combination helps hemiplegic patients regain balance, gait, and foot control, which are critical for ADL such as walking and transferring (60). Studies have shown that patients using a combination of FES and robotic-assisted gait training exhibit faster improvements in walking ability and overall mobility than those using traditional physical therapy alone (61, 62).

#### 4.1.3 Neuroplasticity and cognitive engagement

The combined use of FES, VR, and RAT has also been shown to enhance neuroplasticity. Neuroplasticity is the ability of the brain to reorganize itself by forming new neural connections, which is crucial for recovery after stroke or brain injury. By integrating cognitive tasks through the VR environment while performing physical activities with the RAT and FES, patients strengthened the connection between motor control systems and cognitive function. This multimodal stimulation helps to consolidate the progress made in each session, leading to more lasting improvements (63, 64).

#### 4.1.4 Flexibility of individualized rehabilitation plans

Another notable advantage of combined therapies is the flexibility to tailor rehabilitation programs to the specific needs of each patient. For example, a patient with greater muscle control difficulties may benefit from more frequent FES sessions, while another patient with severe cognitive-motor integration problems may require more VR-based training (65). This flexibility allows clinicians to design individualized rehabilitation programs that maximize ADL recovery potential, making combined therapies a highly adaptable and patient-centered approach.

### 4.2 Challenges of long-term effectiveness and sustainability of modern therapies

While modern rehabilitation therapies such as VR, RAT, and FES have shown significant short-term efficacy, their long-term effectiveness and sustainability remain topics of debate. Over time, these therapies face multiple challenges in sustaining functional recovery, including diminished effectiveness, patient compliance issues, and high equipment costs. This section explores the long-term adaptability of these therapies, analyzes potential sustainability challenges, and suggests future directions for research and development to ensure their long-term efficacy.

#### 4.2.1 Clinical evidence: the debate on long-term efficacy

Although VR, RAT, and FES have demonstrated significant short-term efficacy, their long-term effectiveness remains a topic of debate. Several studies have shown that patients using these modern therapies maintain high ADL function after several months of treatment (66). For example, VR not only helped patients maintain motor function through ongoing cognitive engagement, but also reinforces neural plasticity (35). However, other studies indicate that without continued instruction or reinforcement, the benefits of these interventions tend to decline within 6 to 12 months post-therapy (67). These findings highlight the variability in long-term outcomes, underscoring the need for additional longitudinal studies to assess their sustained impact.

In contrast, behavior-oriented rehabilitation approaches, including CIMT and BCT, have demonstrated sustained real-world benefits beyond structured rehabilitation sessions. Long-term follow-up studies have reported that CIMT can maintain functional gains for up to 2 years, even in chronic stroke patients, due to its focus on task-oriented training and forced use of the affected limb (30). Similarly, BCT has been shown to enhance long-term adherence to physical activity by integrating goal-setting strategies and remote monitoring, leading to higher daily activity levels at 12-month follow-up (17).

A key distinction between these approaches lies in patient adherence. While VR, RAT, and FES provide structured rehabilitation environments, their long-term success often depends on continuous access to technology and therapist supervision. In contrast, CIMT and BCT focus on promoting sustained behavioral change, enabling patients to integrate rehabilitative activities into daily life, even in the absence of direct supervision.

Given these differences, a promising avenue for future rehabilitation strategies may involve integrating behavioral rehabilitation approaches with modern technological interventions. By incorporating BCT principles such as remote feedback and self-monitoring into VR-based rehabilitation, or using CIMT frameworks within robotic-assisted therapy, long-term adherence and functional independence could be significantly improved. Further research should explore how combining behavior-oriented and technology-driven rehabilitation can optimize long-term ADL recovery.

#### 4.2.2 Long-term adaptability of functional electrical stimulation

FES, a therapy designed to restore motor control by re-educating muscles, has been shown to be effective in the short term, particularly in patients with muscle atrophy or impaired motor neurons. Although FES is effective during acute rehabilitation, questions remain about its long-term adaptability and effectiveness. Some studies have suggested that patients may develop tolerance to the stimulation over time, leading to diminished effectiveness (56). Therefore, future research should focus on developing long-term FES protocols that remain effective during different stages of recovery.

#### 4.2.3 Sustainability challenges of robotic-assisted therapy

In the case of RAT, the main challenge for long-term use is the complexity and cost of the equipment. Although RAT can provide precise, repetitive motor training to help restore upper and lower extremity function in the short term, long-term reliance on these devices is often limited by their high cost and accessibility (68). In addition, once treatment has ended, patients may become less dependent on the devices, leading to decreased rehabilitation outcomes. Therefore, the long-term success of RAT depends on the development of more affordable home-use devices and scalable solutions.

#### 4.2.4 Patient compliance and sustainability issues

Another crucial factor in the long-term effectiveness of these therapies is patient compliance. Modern interventions such as VR, RAT, and FES often require frequent and repetitive treatments, which may be difficult for many patients to maintain over time. Studies have shown that patient motivation, psychological factors, and family support significantly influence the long-term success of these interventions (69, 70). Future treatment plans should focus on enhancing patient compliance, perhaps by making VR rehabilitation more engaging through gamification or by simplifying RAT devices for home use.

## 5 Future development of hemiplegia rehabilitation and its significance

The field of hemiplegia rehabilitation is undergoing significant changes as science and technology continue to advance. Future trends in rehabilitation will focus on personalized treatment, remote rehabilitation management, integration of biomedicine and technology, advances in regenerative medicine, and the comprehensive integration of psycho-social rehabilitation. These cutting-edge approaches are expected to significantly enhance the precision and efficiency of rehabilitation, particularly by utilizing personalized plans, remote monitoring, artificial intelligence, big data analysis, and regenerative medicine techniques to accelerate patient recovery. This section will explore these trends and their far-reaching implications for the future development and application of hemiplegia rehabilitation.

### 5.1 Development of personalized rehabilitation therapy

Personalized therapy is one of the core trends in the future hemiplegia rehabilitation. This approach will utilize individual patient data, such as genomics, specific types of neurological damage, rehabilitation potential, and socioeconomic background, to develop tailored rehabilitation plans. Advances in gene therapy and precision medicine have provided insights into the potential for personalized neurological repair (71). Additionally, emerging technologies, such as virtual reality, robotic-assisted devices, and electrical stimulation therapies, hold promise for delivering individualized treatment and optimizing rehabilitation strategies (72). However, despite these advancements, the clinical application of personalized rehabilitation remains limited due to the experimental nature of many treatments, the lack of large-scale validation, and high implementation costs. Further research is needed to establish cost-effective, evidence-based approaches that can enhance accessibility and optimize patient outcomes across diverse healthcare settings.

### 5.2 Popularization of remote and home-based rehabilitation devices

Remote rehabilitation technologies and home-based rehabilitation devices are expected to play a crucial role in the future of post-stroke and motor function recovery. With advances in 5G, wearable sensors, and the Internet of Things (IoT), patients can perform rehabilitation exercises at home while receiving real-time remote monitoring and guidance from healthcare professionals (73). The IoT refers to a network of interconnected smart devices—including wearable motion sensors, home-based robotic assistive devices, and cloud-based rehabilitation platforms—that collect and transmit real-time patient movement data, enabling remote assessment and personalized therapy adjustments. These technologies offer potential benefits, such as improving rehabilitation accessibility, reducing hospitalization costs, and allowing for more continuous, data-driven treatment plans.

However, despite these advancements, significant limitations remain, particularly in lower extremity rehabilitation and gait training. Remote rehabilitation systems often struggle to support long-distance ambulation training, as most home-based programs are limited to stationary exercises or short-distance walking within confined spaces. Camera-based monitoring presents challenges in capturing full-body movements, particularly for tasks such as stair climbing or complex gait patterns, which are essential for real-world mobility (33).

Another critical concern is safety and treatment standardization. Unlike in-clinic rehabilitation, patients engaging in home-based therapy may lack immediate physical assistance, increasing the risk of falls, especially for individuals with severe mobility impairments. Furthermore, the effectiveness of remote rehabilitation remains uncertain in the long term, as patient adherence, digital literacy, and variations in home environments can affect treatment outcomes.

Future research should focus on developing standardized protocols, integrating wearable motion tracking for continuous gait monitoring, and enhancing real-time biofeedback mechanisms to overcome these limitations. Combining behavior-oriented rehabilitation strategies (such as BCT) with remote monitoring systems may further improve patient adherence and long-term functional gains in home-based rehabilitation.

### 5.3 Integration of artificial intelligence and big data

The application of artificial intelligence (AI) and big data analysis in hemiplegia rehabilitation has tremendous potential. Through machine learning and AI algorithms, patient data during rehabilitation (e.g., motion data, brain electrical activity, and electromyography signals) can be analyzed and interpreted to generate personalized rehabilitation recommendations (74). Additionally, AI technology can monitor patients’ recovery progress in real-time, predict rehabilitation trajectories, and adjust the intensity or content of therapy to improve outcomes (75). For example, AI-assisted robotic devices can automatically adjust the intensity of training based on the patient’s performance to prevent overexertion or improper training. The integration of these technologies is expected to make hemiplegia rehabilitation more precise and efficient. However, the clinical implementation of AI-driven rehabilitation remains challenging due to its reliance on high-quality, diverse training datasets, concerns over data privacy and ethical considerations, and the need to maintain patient-therapist interaction for effective rehabilitation. Future research should prioritize enhancing AI model reliability, addressing privacy concerns, and ensuring accessibility to diverse patient populations to maximize clinical benefits.

### 5.4 Advances in regenerative medicine and neural repair technologies

Progress in regenerative medicine, particularly stem cell therapy and neural repair technologies, will play an increasingly important role in hemiplegia rehabilitation (76). In recent years, scientists have used stem cell transplantation to promote neuronal regeneration and repair damaged neural pathways (77). In the future, stem cell therapy may be combined with conventional rehabilitation to accelerate neurological recovery, especially to restore ADL function. Additionally, also of interest are neural interface technologies that connect computers directly to the brain. These technologies help patients control external devices and allow partial functional recovery even in cases of severe neurological damage. However, these approaches remain in experimental stages, with challenges related to efficacy, safety, long-term integration, and clinical scalability. Stem cell-based therapies require further validation through large-scale clinical trials, and neural interface technologies need improvements in stability, biocompatibility, and long-term functionality. Future research should focus on addressing these challenges to ensure the safe and effective clinical application of regenerative rehabilitation strategies.

### 5.5 Comprehensive integration of psycho-social rehabilitation

With growing recognition of the psychological and social aspects of hemiplegia recovery, efforts have been made to incorporate psycho-social rehabilitation into treatment programs. Virtual reality (VR) technology and remote psychological counseling have been explored as potential tools to support mental health, emotional regulation, and social reintegration (78). Augmented reality (AR) and VR-based simulations may offer opportunities for patients to practice real-life scenarios, facilitating adaptation to social environments. However, the clinical integration of psycho-social rehabilitation remains limited, as many interventions lack standardized protocols, robust long-term validation, and consistent patient adherence. Engagement with these programs varies based on cognitive function, motivation levels, and access to mental health resources, which can impact their effectiveness. Moreover, the effectiveness of VR and digital interventions in psycho-social rehabilitation remains under investigation, with mixed results in different patient populations. Future research should prioritize developing standardized, evidence-based psycho-social rehabilitation frameworks, refining digital interventions to enhance patient engagement, and conducting large-scale longitudinal studies to assess their long-term impact on functional and psychological recovery.

## 6 Conclusions and future perspectives

Modern rehabilitation technique such as VR, RAT, and FES offer promising approaches for enhancing (ADL) recovery in hemiplegic patients by providing more personalized, engaging, and technology-driven therapies. However, their long-term effectiveness, generalizability, and clinical integration remain uncertain, requiring further rigorous evaluation. Future research should focus on long-term outcome validation, refining intervention protocols, optimizing multimodal therapy integration, and addressing patient-specific variability. Additionally, improving the cost-effectiveness and accessibility of these technologies is essential to facilitate equitable implementation. While these advancements hold potential, their widespread clinical adoption depends on further evidence-based validation, enhanced accessibility, and ongoing refinement to align with the diverse needs of hemiplegic patients.
